# Long-Term Use of Metformin Is Associated With Reduced Risk of Cognitive Impairment With Alleviation of Cerebral Small Vessel Disease Burden in Patients With Type 2 Diabetes

**DOI:** 10.3389/fnagi.2021.773797

**Published:** 2021-10-29

**Authors:** Zhenjie Teng, Jing Feng, Qianqian Qi, Yanhong Dong, Yining Xiao, Xiaohua Xie, Nan Meng, Huifang Chen, Wenhui Zhang, Peiyuan Lv

**Affiliations:** ^1^Department of Neurology, Hebei Medical University, Shijiazhuang, China; ^2^Department of Neurology, Hebei General Hospital, Shijiazhuang, China; ^3^Department of Endocrinology, Hebei General Hospital, Shijiazhuang, China; ^4^Memory Clinic, Hebei General Hospital, Shijiazhuang, China

**Keywords:** metformin, cerebral small vessel disease, cognitive impairment, diabetes, relationship

## Abstract

**Objective:** Type 2 diabetes (T2D) is a risk factor for cognitive impairment and cerebral small vessel disease (CSVD). The relation of metformin use and cognitive impairment or CSVD is not clear. The objective of this study was to investigate the cross-sectional effects of long-term use of metformin on total CSVD burden and cognitive function in patients with T2D.

**Methods:** A total of 234 participants with T2D from the memory clinic in Hebei General Hospital were enrolled in this retrospective study. Duration of metformin use and dosage were recorded. Along with cerebral magnetic resonance imaging (MRI) examination, Mini-Mental State Examination (MMSE) was also performed to assess their cognitive status. We determined the validated total CSVD score (ranging from 0–4) by combining four markers of CSVD that were visually rated. We used binary logistic regression analysis, ordinal logistic regression analysis and mediation analysis to assess the relation of long-term use of metformin with CSVD burden and cognitive function.

**Results:** Binary logistic regression analysis showed long-term use of metformin was associated with reducing the risk of cognitive impairment (OR: 0.446; 95% Confidence Interval (CI): 0.249 to 0.800; *P* = 0.007), after adjustment of potential confounders, such as total CSVD burden score, age, HbA1c, hypertension, history of stroke, homocysteine, body mass index, TG and HDL-C. Ordinal logistic regression analysis suggested that long-term use of metformin was associated with alleviation of total CSVD burden score (OR: 0.583; 95% CI: 0.359 to 0.943; *P* = 0.027), after adjusting for age, HbA1c, hypertension, history of stroke, homocysteine, body mass index, TG and HDL-C. Mediation analysis showed significant mediation by the presence of severe CSVD burden score for long-term use of metformin in relation to cognitive impairment.

**Conclusion:** Long-term use of metformin was associated with lower rates of cognitive impairment and lower total CSVD burden score in patients with T2D. A proportion of the relation between long-term use of metformin and cognitive impairment may be attributable to alleviation of CSVD burden.

## Introduction

Cognitive impairment is a growing problem, which can range in severity from mild cognitive impairment to dementia. Due to an increase in population aging and growth, the number of people with cognitive impairment is rising, which imposes a tremendous burden on families and society (GBD 2016 Dementia Collaborators, 2019; Hamilton et al., 2021).

Cerebral small vessel disease (CSVD) is a primary cause of cognitive impairment in the elderly and it accounts for about 50% of dementias worldwide (Wardlaw et al., 2019; Zlokovic et al., 2020). CSVD describes pathological processes affecting the small arteries, arterioles, capillaries and small veins of the brain. Signs of CSVD on conventional magnetic resonance imaging (MRI) include white matter hyperintensities (WMH), lacune of presumed vascular origin, cerebral microbleeds (CMBs), brain atrophy, microinfarcts and enlarged perivascular spaces (EPVS) (Wardlaw et al., 2013). CSVD is a dynamic, whole-brain disorder. CSVD burden score, combined the individual markers into one score, can reflect the overall burden and be more representative of CSVD (Staals et al., 2014). Recent studies have revealed that blood-brain barrier (BBB) dysfunction and endothelial cell dysfunction seem to be pivotal factors contributing to the pathogenesis of CSVD (Ihara and Yamamoto, 2016; Wardlaw et al., 2019). Factors, such as oxidative stress, inflammation, hypoxia, and hypoperfusion may lead to BBB and endothelial dysfunction (Grochowski et al., 2018; Low et al., 2019; Zanon et al., 2021), which is worse in patients with cognitive impairment (Nation et al., 2019).

Type 2 diabetes (T2D) is also an important risk factor for cognitive impairment, which in turn can impair self-management of T2D (Xue et al., 2019; Launer, 2020). Metformin, a biguanide derivative, broadly used for treating T2D, has caught considerable attention among researchers because of its ability to improve cognitive function in patients with T2D (Zhang et al., 2020; Gorgich et al., 2021; Kodali et al., 2021). The mechanisms include ameliorating oxidative stress, inhibiting inflammation, mediating nutrient-sensing pathway and enhancing autophagy, although the relationships among these mechanisms are complex and not yet fully understood (Gorgich et al., 2021; Hu et al., 2021; Kodali et al., 2021). However, some studies have implied that metformin may increase the risk of cognitive impairment in patients with T2D (Moore et al., 2013; Porter et al., 2019).

To the best of our knowledge, few studies have investigated the relationship between CSVD burden and metformin use, although metformin could act on some of the mechanisms that lead to CSVD. Collectively, the present study aims to analyze the relation of long-term use of metformin with CSVD burden and cognitive function in patients with T2D.

## Materials and Methods

### Participants

This is a case-control study of patients with T2D from the memory clinic in Hebei General Hospital. From January 2017 to January 2019, a total of 234 patients with a diagnosis of T2D who underwent complete MRI sequences necessary for evaluating CSVD markers were included. Inclusion criteria for all participants were able to undergo cognitive testing and independent in daily life. We excluded those with any contraindication to MRI, history of T2D <6 years, acute stroke or other neurologic disorders (epilepsy, trauma, malignancy, schizophrenia, central nervous system demyelinating diseases, or depressive disorder), other diseases, such as hyperthyroidism, hypothyroidism, severe hepatorenal dysfunction, hematological diseases and carbon monoxide poisoning.

### Clinical Assessment

In this study, the following variables were collected: demographic profiles (age, gender, years of education, height, weight), medical history (hypertension, T2D, coronary heart disease, stroke and intracranial hemorrhage), cigarette smoking and alcohol drinking status and the use of medications. Laboratory markers such as fasting plasma glucose (FPG), hemoglobin A1c (HbA1c), total cholesterol (TC), triglyceride (TG), low density lipoprotein cholesterol (LDL-C), high density lipoprotein cholesterol (HDL-C), very low density lipoprotein cholesterol (VLDL-C), homocysteine and Uric Acid were recorded.

### Metformin Use

All medications, duration of use, and dosage were recorded, which included the ascertainment of metformin usage. Participants were classified into the two groups by duration of metformin usage: (i) duration of metformin use as 6 or less years; and (ii) duration of metformin use as more than 6 years (Ng et al., 2014).

### Magnetic Resonance Imaging Data Acquisition, Interpretation, and Evaluation

Magnetic resonance imaging examination was performed in all patients using 3.0 tesla MR scanners (Signa, GE Healthcare of American). The standardized neuroimaging protocol included T1-weighted imaging (T1WI), T2-weighted imaging (T2WI), fluid-attenuated inversion recovery (FLAIR) and susceptibility weighted imaging (SWI). MR scanning parameters: T1WI: repetition time (TR)/echo time (TE) = 1,909/20.2 milliseconds (ms), slice thickness = 5 mm; T2WI: TR/TE = 5,000/125 ms, slice thickness = 5 mm; FLAIR: TR/TE = 8,502/159.4 ms, slice thickness = 5 mm; and SWI: TR/TE = 78.6/47.6 ms, slice thickness = 2 mm.

Imaging markers of CSVD were evaluated by two readers (QQ and YX) according to the Standards for Reporting Vascular Changes on Neuroimaging (STRIVE) criteria (Wardlaw et al., 2013). WMH of presumed vascular origin displayed as bilateral, mostly symmetric hyperintensities on T2WI and FLAIR. The periventricular WMH (range 0–3) and the deep-WMH (range 0–3) were rated according to Fazekas rating scale (Fazekas et al., 1993). A lacune of presumed vascular origin was defined as a round/ovoid-shaped, fluid-filled (similar signal as CSF) cavity measuring 3–15 mm in diameter on T1WI or T2WI sequences, following the territory of a perforating arteriole. The presence, number, and location of the lacune were recorded. CMBs were defined as a small area of signal void (smaller than 10 mm) on SWI and rated using the Microbleed Anatomical Rating Scale (Gregoire et al., 2009). EPVS was defined as round, oval, or linear lesions with CSF-like signals measuring <3 mm on T2WI without a hyperintense rim, and they were rated on a previously described semi-quantitative scale from 0 to 4 (Doubal et al., 2010). In case of disagreement on any of the markers, a third reader (HC) assessed the images in order to achieve consensus. All ratings were performed blinded to all patient data.

On the 4-point CSVD burden scale, one point was allocated to each of the following MRI parameters: severe WMH (periventricular WMH Fazekas score 3 or deepWMH Fazekas score 2 or 3), presence of one or more acunes of presumed vascular origin, one or more deep CMBs, and moderate to severe (grade 2–4) basal ganglia EPVS. Thus, the total CSVD burden score could range from 0 to 4 (Staals et al., 2015; Jiang et al., 2019). Severe CSVD burden score was defined when score >2.

### Cognitive Function Assessment

All subjects underwent cognitive evaluation using the translated version of the Mini-Mental State Examination (MMSE), which has been validated for use in Chinese adults. Cognitive impairment was determined from the optimal cut-off points based on years of education. The optimal cut-off points for cognitive impairment screening were 16/17 for illiterate (sensitivity 87.6% and specificity 80.8%), 19/20 for individuals with 1–6 years of education (sensitivity 93.6% and specificity 92.7%), and 23/24 for individuals with 7 or more years of education (sensitivity 94.3% and specificity 94.3%) (Li et al., 2016).

### Statistical Analyses

Continuous variables were presented with mean (standard deviation) or median (interquartile range) as appropriate. Categorical variables were presented with case (percentage). Mann-Whitney *U* tests or *T* tests were used to analyze continuous variables, and *χ^2^* tests were used for categorical variables. The total CSVD burden was used as an ordinal variable, which was analyzed by Mann-Whitney *U* tests. Binary logistic regression was performed to investigate the relations of multiple clinical and imaging variables on cognitive impairment. Ordinal logistic regression was used to investigate the effect of multiple variables on CSVD burden. The proportional odds assumption was checked through the test of parallel lines and no violations were identified. Values of *P* < 0.05 (two-sided) were considered statistically significant. The SPSS software package 21.0 (IBM corporation, Armonk, NY, United States) was used for all these analytic processes.

Mediation analysis was performed in R, version 4.1.0 (R Foundation for Statistical Computing, Vienna, Austria) using Mediation Package, version 4.5.0 to assess whether the presence of CSVD mediates the relation between long-term use of metformin and cognitive impairment. Logistic regression models were used to regress the mediator (CSVD burden score >2; dichotomous) on X (long-term use of metformin; dichotomous) and to regress Y (cognitive impairment; dichotomous) on mediator and X. Mediation analyses were repeated, allowing for an interaction between X and the mediator.

## Results

### Participants Characteristics

This study included 234 patients with T2D. Mean age of the study participants was 67.8 ± 8.9 years and 53.8% (*n* = 126) were male. All individuals were divided into cognitive impairment (CI) (*n* = 101) group and no cognitive impairment (NCI) (*n* = 133) group according to cognitive function assessment. Characteristics of the study participants between CI and NCI group are shown in Table 1. The participants in the CI group were significantly older and more likely to have a history of stroke than those in the NCI group. The CI group presented higher HbA1c and homocysteine levels, but shorter duration of metformin use than NCI group. In addition, the total CSVD burden score was higher in subjects with CI.

**TABLE 1:** Characteristics of the study participants between CI and NCI group.

Variable	CI group (*n* = 101)	NCI group (*n* = 133)	*P* value
Age, mean (SD), year	70.2 ± 8.4	66.0 ± 8.9	**< 0.001**
Sex (male), n (%)	57 (56.4)	69 (51.9)	0.489
Body mass index, mean (SD), kg/m^2^	24.8 ± 9.6	25.4 ± 3.4	0.123
Current smoking, n (%)	39 (38.6)	45 (33.8)	0.450
Alcohol use, n (%)	26 (25.7)	44 (33.1)	0.225
Hypertension, n (%)	80 (79.2)	94 (70.7)	0.139
Coronary heart disease, n (%)	30 (29.7)	47 (35.3)	0.364
History of stroke	55 (54.5)	43 (32.3)	**0.001**
Duration of T2D, median (IQR), year	10 (7–17)	10 (8–14)	0.462
FPG, median (IQR), mmol/L	7.17 (5.76–9.42)	6.95 (5.86–8.46)	0.631
HbA1c, median (IQR), %	7.40 (6.70–8.19)	7.00 (6.40–7.85)	**0.023**
Metformin use (>6 years), n (%)	35 (34.7)	75 (56.4)	**0.001**
Other anti-diabetic drugs Glitazones, n (%)	31 (30.7)	44 (33.1)	0.698
Dipeptidyl peptidase-4 (DPP-4) inhibitors, n (%)	25 (24.8)	37 (27.8)	0.598
Sulfonylureas, n (%)	42 (41.6)	65 (48.9)	0.268
Insulin, n (%)	55 (54.5)	62 (46.6)	0.235
TC, median (IQR), mmol/L	4.45 (3.56–5.21)	4.25 (3.60–5.27)	0.998
TG, median (IQR), mmol/L	1.61 (1.12–2.16)	1.33 (1.08–2.09)	0.124
HDL-C, median (IQR), mmol/L	1.04 (0.87–1.22)	1.06 (0.93–1.28)	0.148
LDL-C, median (IQR), mmol/L	2.75 (2.02–3.45)	2.69 (2.20–3.52)	0.860
VLDL-C, median (IQR), mmol/L	0.45 (0.29–0.67)	0.45 (0.31–0.63)	0.759
Uric Acid, median (IQR), umol/L	305 (234–378)	290 (253–355)	0.932
Homocysteine, median (IQR), umol/L	15.5 (12.9–19.2)	14.2 (11.6–16.2)	**< 0.001**
Total CSVD burden score			**< 0.001**
0, n (%)	8 (7.9)	36 (27.1)	
1, n (%)	16 (15.8)	31 (23.3)	
2, n (%)	15 (14.9)	28 (21.1)	
3, n (%)	30 (29.7)	26 (19.5)	
4, n (%)	32 (31.7)	12 (9.0)	

*Bold values indicates *P* < 0.05. CI, cognitive impairment; NCI, no cognitive impairment; SD, standard deviation; IQR, interquartile range; FPG, fasting plasma glucose; HbA1, hemoglobin A1c; TC, total cholesterol; TG, triglyceride; LDL-C, low density lipoprotein cholesterol; HDL-C, high density lipoprotein cholesterol; VLDL-C, very low density lipoprotein cholesterol.*

### Long-Term Use of Metformin and Cognitive Impairment

**FIGURE 1:**
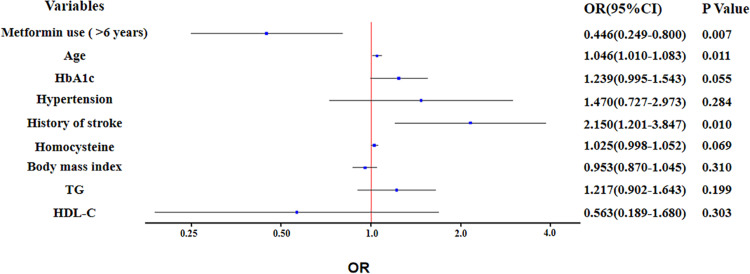
Binary logistic regression analysis for the associated factors with cognitive impairment.

The proportion of participants with metformin use of >6 years was significantly lower in CI group than NCI group (*P* = 0.001) (Table 1). In unadjusted binary logistic regression, long-term use of metformin was associated with reducing the risk of cognitive impairment (OR: 0.410; 95% Confidence Interval: 0.240 to 0.700; *P* = 0.001). The association remained significant (OR: 0.446; 95% Confidence Interval (CI): 0.249 to 0.800; *P* = 0.007), after further adjustment for potential confounders, such as total CSVD burden score, age, HbA1c, hypertension, history of stroke, homocysteine, body mass index, TG and HDL-C (Figure 1).

### Long-Term Use of Metformin and Total Cerebral Small Vessel Disease Burden Score

**FIGURE 2:**
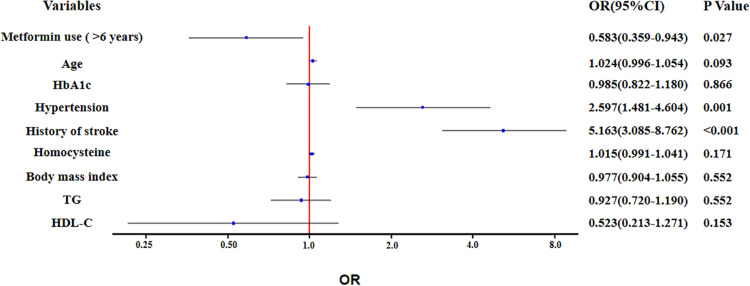
Ordinal logistic regression analysis for the associated factors with total cerebral small vessel disease (CSVD) burden.

In unadjusted ordinal logistic regression analysis, long-term use of metformin was associated with alleviation of total CSVD burden score (OR: 0.511; 95% Confidence Interval: 0.322 to 0.089; *P* = 0.004). This trend remained statistically significant (OR: 0.583; 95% Confidence Interval: 0.359 to 0.943; *P* = 0.027), after adjusting for age, HbA1c, hypertension, history of stroke, homocysteine, body mass index, TG and HDL-C (Figure 2).

### Mediation by Severe Cerebral Small Vessel Disease Burden Score

**FIGURE 3:**
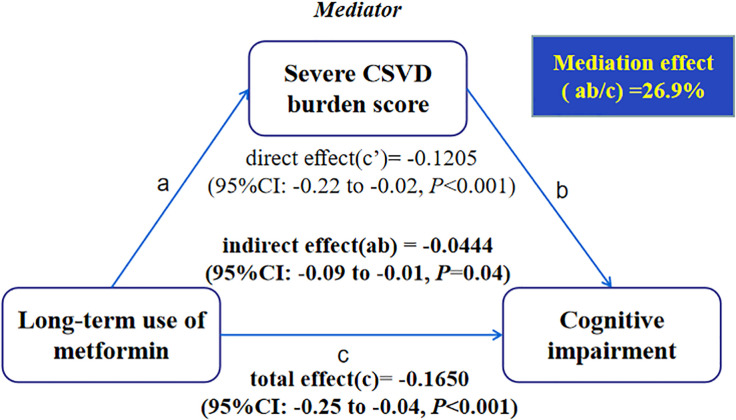
Causal mediation analysis is shown for the presence of severe CSVD burden score as a mediator in the relation between long-term use of metformin and cognitive impairment.

Mediation analysis showed significant mediation by the presence of severe CSVD burden score for long-term use of metformin in relation to cognitive impairment after controlling the regression analyses for age, hypertension, history of stroke and body mass index (Figure 3). In the relation between long-term use of metformin and cognitive impairment, 26.9% of the total effect was attributable to mediation by the presence of severe CSVD burden score (*P* = 0.04). Conclusions did not change when the possible interaction between long-term use of metformin and CSVD burden score was taken into account.

## Discussion

In this cross-sectional study, we investigated the association of long-term use of metformin with total CSVD burden and cognitive function in patients with T2D. To our knowledge, this is the first study exploring the association between long-term use of metformin and total CSVD burden. We found that long-term use of metformin was a protective factor for total CSVD burden and cognitive impairment after controlling for several confounders. Moreover, the presence of severe CSVD burden score was a significant mediator in the relation between long-term use of metformin and cognitive impairment. Furthermore, although no temporal relations can be derived from this cross-sectional study, mediation analysis supports the hypothesis that long-term use of metformin may alleviate the total CSVD burden, which, in turn, may reduce the risk of cognitive impairment.

Compared to previous studies, an important strength of our study was the assessment of the relationship between CSVD burden and metformin use in patients with T2D. In our study, long-term use of metformin was associated with lower total CSVD burden score, which suggested that long-term use of metformin may delay the progression of CSVD. The current study adds to the literature supporting that metformin may have a neuroprotective effect. Although the mechanism remains elusive, several possible mechanisms may explain this effect. One possible mechanism of metformin induced protection in CSVD is chronic activation of AMP-activated protein kinase (AMPK), which plays important roles in regulation of energy metabolism (Steinberg and Carling, 2019; Crunkhorn, 2021). AMPK is highly expressed in neurons, neuroglia and astrocytes of the neurogliovascular unit. Interestingly, several of the cellular and functional constituents of the neurogliovascular unit represent possible entry points for disease mechanisms in CSVD (Wardlaw et al., 2019). Increasing evidence is showing that AMPK activation alleviates the damage of BBB and improves endothelial functions (Zhao et al., 2014; Prasad et al., 2017; Wang et al., 2017; Wu et al., 2020), which were key factors contributing to the pathogenesis of CSVD (Wardlaw et al., 2019). As a well-recognized AMPK activator, a previous study of experimental animal models found that metformin could inhibit inflammation, thereby alleviating endothelial injury and lowering BBB permeability by an AMPK-dependent intercellular adhesion molecule-1 (ICAM-1) down-regulation (Liu et al., 2014). Additionally, lines of evidence suggested AMPK activation plays a key role of metformin in improving endothelial functions (Salvatore et al., 2020). Besides, chronic metformin treatment could enhance angiogenesis by AMPK signaling and then offer potent neuroprotective effects (Venna et al., 2014). Other possible mechanisms, such as ameliorating oxidative stress (Zeng et al., 2019), inhibiting inflammation (Jin et al., 2014) or other neuroprotective mechanisms may some also partly explain that metformin may delay the progression of CSVD (Grochowski et al., 2018; Wardlaw et al., 2019). Therefore, these above mechanisms may plausibly explain the result in our study that long-term use of metformin was associated with alleviation of total CSVD burden.

Previous studies have mainly focused on the association between metformin use and cognitive impairment. A meta-analysis, including 10 studies, suggested that metformin use significantly reduced the occurrence of cognitive impairment in patients with T2D (Zhang et al., 2020). Similarly, two recent animal studies also found metformin can improve cognitive function (Gorgich et al., 2021; Kodali et al., 2021). Whereas, Porter et al. (2019) reported that metformin use of older adults was associated with an increased risk (34 to 36%) of cognitive impairment. In addition, the Diabetes Prevention Program Outcomes Study (DPPOS) found that exposure to metformin was not related to cognition (Luchsinger et al., 2017). The discrepancy may be attributable to duration of metformin usage and T2D, education levels, economic differences and other related confounders. Consistent with our findings, a prospective observational study conducted by Samaras et al. (2020) confirmed that metformin use over 6 years in older people with T2D was related with lesser decline in global cognition and executive function and lower dementia risk compared with those not receiving metformin. However, CSVD, as an important cause of cognitive impairment, should be taken into account when explored the relationship between metformin use and cognitive impairment in researches, yet few do. In our study, after controlling for total CSVD burden score and other confounders, we still found metformin use over 6 years was associated with reducing the risk of cognitive impairment. Furthermore, our result suggest that a proportion of the association may be attributable to alleviation of CSVD burden after controlling the regression analyses for age, hypertension, history of stroke and body mass index in mediation analysis.

Our study has some limitations. Firstly, the cross-sectional design allows the investigation of only associations. Secondly, the study was conducted in one center with a relatively small sample size and the generate sampling bias can not be excluded. However, the study assessed four markers of CSVD instead of focusing on one or two. Thirdly, metformin usage and duration of its usage were recorded by self-reports of participants instead of medical records. Furthermore, MMSE is a measure of global cognitive function. Therefore, our research could not explore the effects of metformin on specific cognitive domains. Finally, some other potential confounders, such as diabetes severity, were not taken into account and may bias our analyses. Future large longitudinal studies will be needed to address these issues.

## Conclusion

In conclusion, long-term use of metformin was associated with lower rates of cognitive impairment and lower total CSVD burden score in patients with T2D. We demonstrated the likelihood of the protection by metformin on cognitive function and CSVD. More importantly, a proportion of the beneficial effects of long-term use of metformin on cognitive function may be attributable to alleviation of CSVD burden. Therefore, we believe metformin has the potential to be useful in the clinical treatment of cognitive impairment and CSVD. To fully assess the mechanisms of beneficial effects of metformin on cognitive function and CSVD, future longitudinal and randomized studies are needed.

## Data Availability Statement

The original contributions presented in the study are included in the article/supplementary material, further inquiries can be directed to the corresponding author.

## Ethics Statement

The studies involving human participants were reviewed and approved by the Ethical Committee of Hebei General Hospital. Written informed consent for participation was not required for this study in accordance with the national legislation and the institutional requirements.

## Author Contributions

PL designed the study protocol and revised the manuscript. ZT and JF wrote the manuscript. YD and HC contributed to acquisition and interpretation of data. QQ, YX, XX, NM, and WZ made substantial contributions to data acquisition and analysis. All authors contributed to the article and approved the submitted version.

## Conflict of Interest

The authors declare that the research was conducted in the absence of any commercial or financial relationships that could be construed as a potential conflict of interest.

## Publisher’s Note

All claims expressed in this article are solely those of the authors and do not necessarily represent those of their affiliated organizations, or those of the publisher, the editors and the reviewers. Any product that may be evaluated in this article, or claim that may be made by its manufacturer, is not guaranteed or endorsed by the publisher.
